# Ocular manifestations among patients with congenital insensitivity to pain due to variants in *PRDM12* and *SCN9A* genes

**DOI:** 10.1002/ajmg.a.62968

**Published:** 2022-09-16

**Authors:** Baker Elsana, Ahed Imtirat, Ronit Yagev, Libe Gradstein, Pierre Majdalani, Oren Iny, Ruti Parvari, Erez Tsumi

**Affiliations:** ^1^ Ophthalmology Department Soroka University Medical Center and Clalit Health Services (Affiliated with the Faculty of Health Sciences, Ben‐Gurion University of the Negev) Beer Sheva Israel; ^2^ The Shraga Segal Department of Microbiology, Immunology & Genetics, Faculty of Health Sciences Ben‐Gurion University of the Negev Beer‐Sheva Israel; ^3^ The National Institute for Biotechnology in the Negev Ben‐Gurion University of the Negev Beer‐Sheva Israel

**Keywords:** CIP, congenital insensitivity to pain, dry eye syndrome, neurotrophic keratopathy, *PRDM12*, *SCN9A*

## Abstract

Congenital insensitivity to pain (CIP) is a group of rare genetic disorders with a common characteristic of absent sensation to nociceptive pain. Here we present a series of six patients; three had a novel variant in the *PRDM12* gene (group A), and three had a missense variant in the *SCN9A* gene (group B). We compared the ocular manifestations between the two groups. Records of these patients from 2009 through 2018 were reviewed. The retrieved data included demographics, genetic analysis results, ocular history and ophthalmic findings including visual acuity, corneal sensitivity, tear production, ocular surface findings, cycloplegic refraction, and fundoscopy. We found that patients with *PRDM12* variant had more severe manifestations of ocular surface disease, with more prevalent corneal opacities and worse visual acuity, compared to patients with *SCN9A* variant.

## 1 INTRODUCTION

Congenital insensitivity to pain (CIP) is a rare condition that belongs to the group of hereditary sensory and autonomic neuropathies, where affected individuals do not experience any pain throughout their lifetime. CIP patients are prone to self‐inflicted injuries, as well as bone fractures and burns that may cause permanent and devastating disabilities. Intellectual ability and sweat function are preserved (Schon et al., 2020).

Variants in several genes have been found to be associated with CIP. The voltage‐gated sodium channel type IX α subunit *(SCN9A)* variants have been reported to cause insensitivity to pain and anosmia, while other sensory modalities remain intact (Cox et al., 2010). Another gene is the epigenetic regulator PR domain zinc finger protein 12 (*PRDM12*), which is essential for human pain perception and its variants interrupt the development of the Aδ and C nociceptive sensory neurons (Chen et al., 2015).

The ocular manifestations of CIP may include neurotrophic keratopathy, superficial punctate keratitis (SPK), corneal ulcers and opacities, and dry eye syndrome (Mimura et al., 2008). Patients with *PRDM12* variants present with reduced lacrimation, corneal abrasions, and reduced or absent corneal blink reflex, causing keratitis and corneal scarring (Chen et al., 2015; Zhang et al., 2016). The data in the current literature regarding ocular manifestations in patients with *SCN9A* are inconclusive.

In this study, we present a novel variant in the *PRDM12* gene causing CIP. We also describe ocular findings and compare the natural history of the disease in patients with a *PRDM12* and *SCN9A variants*.

## 2 MATERIALS AND METHODS

This retrospective case series included all patients with a confirmed genetic diagnosis of CIP who visited the outpatient clinic at the Soroka University Medical Center (SUMC) from 2009 through 2018. The study was approved by the SUMC Institutional Review Board and Ethics Committee and fully adhered to the tenets of the Declaration of Helsinki.

The genetic analysis of the patients with the *PRDM12* variant (group A) was done by Sanger sequencing of the polymerase chain reaction product obtained using primers F: 5′tgccttacctggtccttgat 3′ and R: 5′ tggtccctacacaacagtgc 3′, annealing temperature 59°C. Patients carrying the *SCN9A* variant (group B) underwent genetic analysis as described by Cox et al. (2010).

Both groups were followed by an ophthalmologist and a pediatrician at the outpatient clinic. Information regarding demographics, medical, and surgical history, and previous hospitalizations due to ocular complications were obtained from electronic medical records. The collected ophthalmic data included Snellen chart visual acuity (VA) measurements at the last follow‐up visit (if not possible, counting fingers method was used), cycloplegic refraction, corneal sensitivity assessments by cotton thread test, presence of corneal ulcers and opacities, as well as superficial punctate keratitis (SPK) which was graded according to the method described by Miyata et al. (2003). The active corneal ulcer was defined as a corneal infiltrate with epithelial defect. Tear production was measured using Schirmer test after instillation of 0.4% oxybuprocaine hydrochloride for local anesthesia and insertion of Schirmer strip (TearFlo®) into the lower fornix. The length of wetting in millimeters was recorded after 5 min. We also collected tear breakup time (TBUT), posterior segment findings, and data from available ancillary exams (e.g., anterior segment photos).

## 3 RESULTS

**FIGURE 1:**
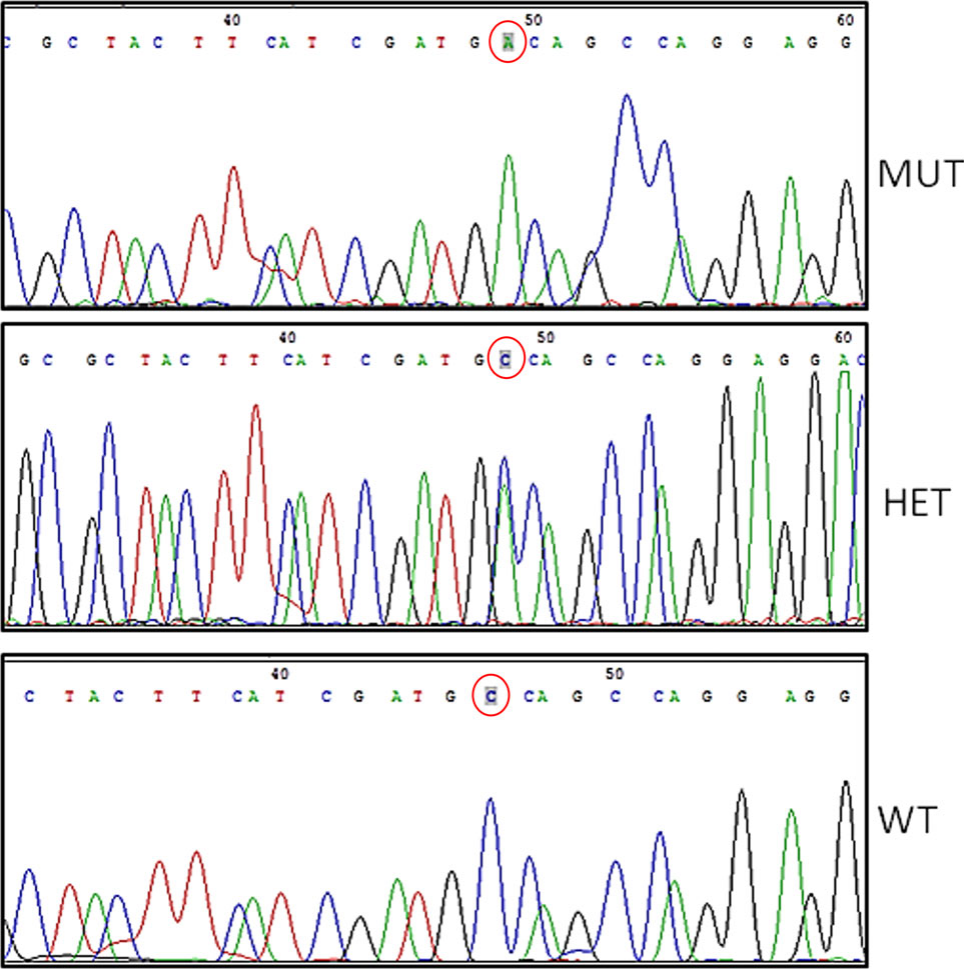
Chromatogram presenting the *PRDM12* sequence of the affected individual CIP3 in comparison to his heterozygous parent and an unrelated normal control subject. HET, heterozygous; MUT, mutation; WT, normal control

Patients' demographic data and clinical findings are summarized in Table 1. Six patients diagnosed genetically with CIP were divided into two groups of three patients each. Patients in group A were homozygous for the *PRDM12* variant: Chr9:133543585(GRCh37/hg19); c455C>A(NM_021619.3); p.Ala152Asp, which has not been described previously. The chromatogram of the sequencing reactions for the affected and control individuals is presented in Figure 1. Group B patients were homozygous for the *SCN9A* variant: R896Q (Cox et al., 2010). The American College of Medical Genetics and Genomics (ACMG) classification for the aforementioned *PRDM12* and *SCN9A* variants were of uncertain significance (Miller et al., 2021). The ACMG evidence for the *PRDM12* variant was PM2 (absent from controls in Exome Sequencing Project, 1000 Genomes Project, or Exome Aggregation Consortium) and for the *SCN9A* variant was PM1 (located in a mutational hot spot and/or critical and well‐established functional domain), PM2 (see above), PP3 (multiple lines of computational evidence such as conservation, evolutionary, and splicing impact support a deleterious effect on the gene or gene product) and BP1 (missense variant in a gene for which primarily truncating variants are known to cause disease). The variant in *SCN9A* was demonstrated to abrogate normal subcellular localization and function of the encoded Na(v)1.7 (Cox et al., 2010). The variant in *PRDM12* was identified in patient CIP3 by exome sequencing which did not suggest any other causative variation. It causes the replacement of a highly evolutionarily conserved amino acid (down to fish, Figure 3) and it is localized to the functional PR/SET domain. Three other variants in this domain were reported to cause CIP (Chen et al., 2015). Moreover, it was not present in the Genome Aggregation Database (gnomAD), neither in our collection of 780 individual Bedouin exomes.

**TABLE 1:** Demographic data and ocular findings of six patients with congenital insensitivity to pain

	Age, years	Gender	Consanguinity	Gene variant	FU (months)	Hosp., days	Eye	Visual acuity	CO	CS	Refraction	SPK grade		TBUT	Schirmer (mm)	Corneal abscess/age (years)	Surgery
												A	D				
1	11	F	+	*PRDM12*	94	4	Right	20/32	−	−	+2.25–3.50 X175	3	3	4	35		LT, PO
							Left	20/63	+	−	+3.50–3.25 X40	3	3	4	35	2, 10	LT, PO
2	3	M	+	*PRDM12*	23	40	Right	FC 1.5 m	+	−	NA	3	3	1	15	1	6AM + 7LT, PO
							Left	FC 1 m	+	−	NA	3	3	1	14	1	5AM + 8LT, 2CCG, PO
3	6	M	NA	*PRDM12*	53	35	Right	20/200	+	NA	NA	NA	NA	NA	NA	1	LT
							Left	20/40	+	NA	+3.25–2.00 X15	NA	NA	NA	NA	2	LT
4	24	F	+	*SCN9A*	143	0	Right	20/200	+	+	−0.75‐4.50 X145	2	3	7	3	1	3LT
							Left	20/32	−	+	+1.00–0.50 X14	1	3	7	7		
5	16	F	+	*SCN9A*	128	0	Right	20/32	−	−	PLANO	0	0	3	<1		
							Left	20/32	−	−	PLANO	0	0	7	<1		
6	13	F	+	*SCN9A*	121	0	Right	20/80	+	+	+5.25–5.00 X40	1	3	2	<1	1	LT
							Left	20/32	−	+	+4.25–1.00 X179	1	3	2	<1		

*Note*: Patients 1 and 2 were siblings, as were patients 4–6. SPK grade represented by area (A) ranging from A0 ‐ no staining to A3 ‐diffuse staining and density (D) ranging from D0‐no staining to D3 lesions are dense and coalescent. Visual acuity refers to BCVA at the last visit.

Abbreviations: AM, amniotic membrane; BCVA, best corrected visual acuity; CCG, corneal covering graft; CO, corneal opacity; CS, corneal sensitivity as assessed by corneal blink reflex; F, female; FC, finger counting; FU, follow‐up; LT, lateral tarsorrhaphy; M, male; NA, not available; PO, punctal occlusion; SPK, superficial punctate keratopathy; TBUT (seconds), tear break‐up time.

Group A included two first‐degree siblings (a girl, age 11 years and a boy, age 3 years; patients CIP1 and CIP2), who were offspring from a consanguineous marriage, and a third unrelated patient (patient CIP3; 6 years old). Group B included three sisters with the same father, but two different mothers (CIP4 and 5 from one mother and CIP6 from a different mother). The patients in group B were described in a previous study (Cox et al., 2010).

Ocular manifestations were the first symptoms of CIP in 4 of the 6 patients: all patients in group A (CIP1, CIP2, and CIP3) and one patient in group B (CIP6). CIP1 presented with red eye and corneal ulcer at the age of 2 years 11 months. CIP2 presented with red eye and ocular secretions at 8 months of age. CIP3 presented with corneal erosions at 1.5 years, and CIP6 presented with red eye and corneal opacities at the age of 1 year. All patients in group A, versus two in group B (CIP4 and 6) developed corneal ulcers. In all of them the ulcers were diagnosed in the first 3 years of life, did not resolve with topical treatment and required lateral tarsorrhaphy. In group A, all patients were hospitalized because of corneal ulcers for various periods at some point in life (on average for 23 days), whereas patients in group B did not need hospitalization and were managed as outpatients. After obtaining cultures from the ulcer, all patients were treated aggressively with topical or subconjunctival fortified antibiotics and with lateral tarsorrhaphy (LT). CIP5 presented at 6 years of age with dry eye complaints and was followed because of dry‐eye syndrome. In group A, CIP2 had a severe course of ocular surface disease. He required LT and amniotic membrane transplantation several times to treat nonhealing corneal ulcers in both eyes. He also underwent tectonic corneal graft transplantations due to corneal melting and descemetocele in his left eye.

**FIGURE 2:**
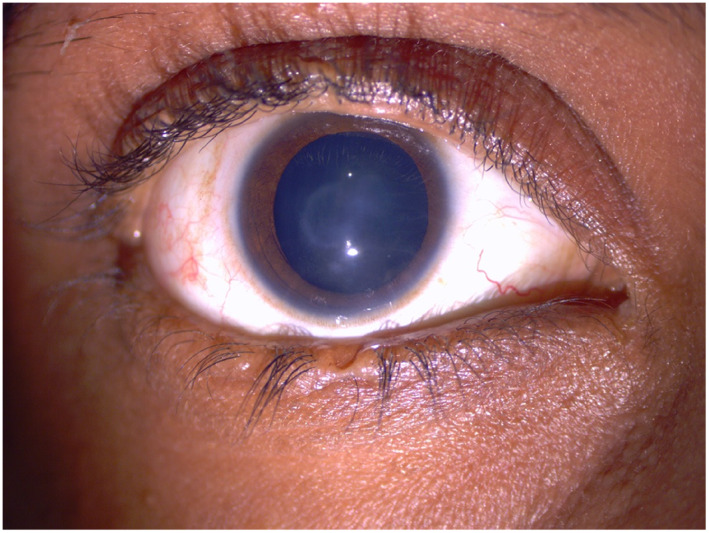
Right eye of patient CIP4 showing central corneal opacity. Note the notch in upper and lower eyelids where tarsorrhaphy was performed

VA and corneal blink reflex results are detailed in Table 1. Corneal opacities were observed in five of six eyes in group A, as compared to two of six eyes in group B (Figure 2).

Most patients in both groups had signs of dry eye. In group A, two siblings, CIP1 and 2, manifested dense and diffuse SPK (A3D3). They required permanent punctal occlusion to treat the ocular surface disease. In group B, the SPK grades were less severe, ranging from A0D0 to A2D3, although Schirmer test measurements yielded decreased tear production, as compared with group A. All patients were managed with frequent instillation of lubricating eye drops.

## 4 DISCUSSION

**FIGURE 3:**
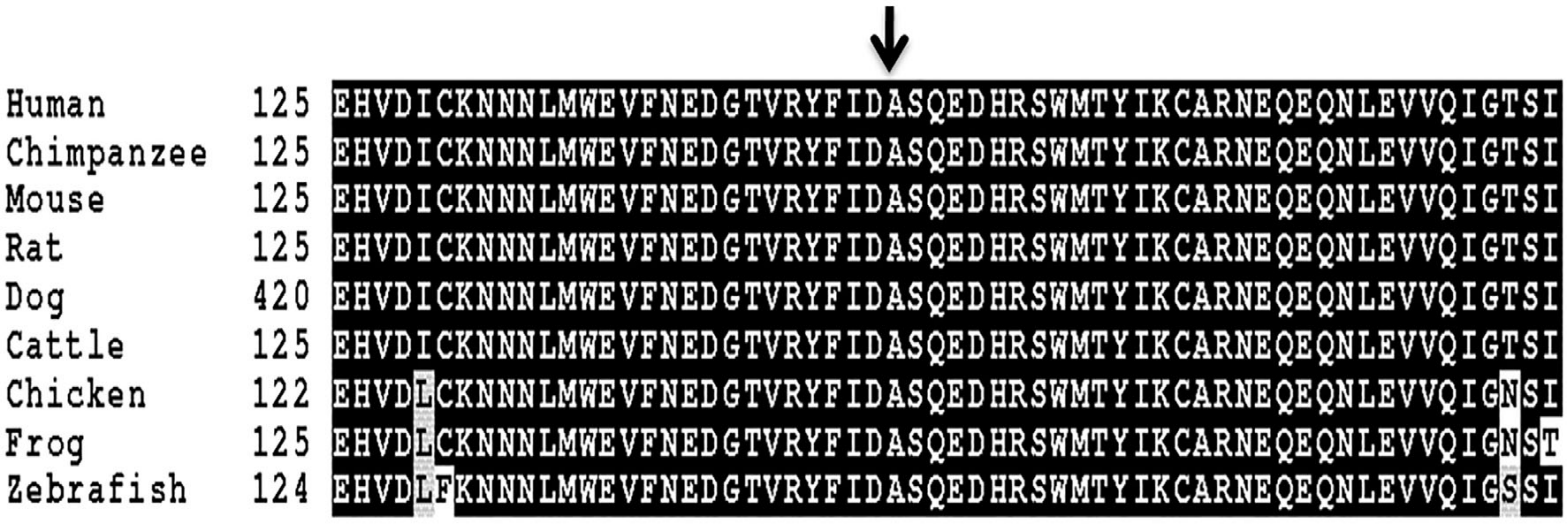
Multiple protein sequence alignment generated with the program Clustal W. Sequence comparison showed that the *PRDM12* missense variant (arrow) observed in patients with CIP affects an amino acid that is identical in *PRDM12* and its orthologs

This case series presents six CIP patients with two genetic variants which correspond to two clinical groups: (A) A developmental disorder where nociceptors fail to develop or undergo early apoptosis due to a lack of trophic signals and (B) Nociceptors have developed and are in their correct anatomical position but are unable to respond to tissue‐damage signals (Drissi et al., 2020). Peripheral sensory nerve biopsies from CIP patients with variants in *PRDM12* (first group) show a diminution of Aδ but not C fibers (Chen et al., 2015). *PRDM12* functions as a transcriptional regulator, which is important in the differentiation from a neural crest cell to a precursor sensory neuron (Drissi et al., 2020). Here, we present a novel variant in the *PRDM12* gene, Chr9:133543585(GRCh37/hg19);c455C>A(NM_021619.3);p.Ala152Asp. Ala at position 152 is highly evolutionarily conserved (Figure 3). It is located in the PR domain in the hydrophobic pocket, which binds the substrate for methylation. The replacement of Ala by the highly charged Asp is predicted to disrupt the hydrophobicity of this pocket and thus, interfere with the binding of this substrate.

In the second group of patients, the ocular manifestations are caused by a variation in *SCN9A*. This gene encodes the protein voltage‐gated sodium channel Nav1.7 acting at the cell membrane of all classes of nociceptors, where it is strongly expressed. It forms a channel that allows only sodium ions to flow through a central pore from the exterior to the cell's interior, with the pore open or closed, depending on the voltage potential across the cell membrane. This makes *SCN9A* a critical and nonredundant element in sensing pain, although it is unclear how this is achieved. The missense variant in our patients has been described previously (Cox et al., 2010) and it was demonstrated to abrogate the channel's subcellular localization and function.

CIP is a rare disorder and little is known about its ocular manifestations. The findings in this series demonstrated that patients with both genetic variants presented with ocular complications of varying severity that were primarily restricted to the ocular surface. They tended to have dry eye signs, as well as corneal ulcers and opacities, which compromised the development of normal vision. These manifestations are likely related to the lack of corneal sensitivity, as was previously suggested (Chen et al., 2015; Elsana et al., 2021; Mimura et al., 2008; Zhang et al., 2016).

In this study, we compared for the first time the ocular manifestations between the CIP patients with *PRDM12* and *SCN9A* variants. We found that patients with *PRDM12* variant (group A) tended to have a poorer ocular surface disease and VA than did patients with *SCN9A* variant (group B). Absent corneal blink reflex, presence of corneal opacities and higher SPK grades were more prevalent among patients in group A. Interestingly, tear production was decreased among all patients in group B. However, it should be noted that both Schirmer test and TBUT may be of limited value for assessment of dry eye signs in children. According to a recent study by Ong Tone et al. (2019), the only reliable method is corneal fluorescein staining.

Refractory, nonhealing corneal ulcers that required surgery were more frequent and tended to be more severe and difficult to treat in group A than in group B. This was demonstrated by an average of 23 days of hospitalization to manage corneal morbidity in group A. In contrast, none of the patients in group B were hospitalized. Similar results regarding corneal blink reflex and corneal opacities were reported in other patients with *PRDM12* variants. In one study of affected individuals from 11 families, corneal insensitivity as evidenced by absent blink response led to progressive corneal scarring in many cases. Affected individuals also tended to have reduced tear function (Chen et al., 2015). In another study of five PRMD12‐CIP patients, 23–57 years of age, all with decreased tear production and absent corneal blink reflex, two had severely impaired vision in one eye (Zhang et al., 2016).

Ocular findings vary among patients with *SCN9A* variants. McDermott et al. (2019) reported that the corneal blink reflex was intact in three patients (31, 34, and 44 years old). In our study, the blink reflex was preserved in two patients and absent in the third one, and all three had decreased lacrimal production. Additional reported patient also did not exhibit corneal blink reflex and developed bilateral sterile corneal ulcers that were attributed to neurotrophic keratopathy (Kim et al., 2015).

All but one patient in this case series developed corneal ulcers, all before the age of 3 years, and in most of them as a first manifestation of the disease. To the best of our knowledge, no studies have addressed the ways to prevent ocular complications in the CIP patients. Based on our experience, we suggest the following measures for management of this condition. Early diagnosis is of paramount importance for prevention of corneal ulcers and the resultant scarring. Children with family history of CIP should undergo a prompt genetic analysis to check for the presence of the familial genetic variant. For patients without a family history, who develop recurrent corneal ulcers in young age, genetic testing for CIP should also be ordered, especially in the presence of other signs that raise the suspicion of CIP, such as inadvertent trauma without manifesting pain, tongue biting, and absence of pain during blood sampling. Once the diagnosis of CIP is established or suspected, a comprehensive ophthalmologic examination, especially of the ocular surface for dry eye signs, corneal irregularities, and opacities, as well as evaluation of corneal sensitivity should be done shortly after birth. It is imperative to educate parents about the ocular manifestations of the disease and the importance of seeking urgent ophthalmic care if alarming signs such as red eye, corneal whitening, or secretions appear. Regular ophthalmologic follow‐up is advised, especially for patients lacking corneal sensitivity. Once ocular surface disease is detected, aggressive treatment should be initiated to prevent the development of corneal opacities and vision loss. For patients with dry eye signs, we recommend abundant ocular lubrication with preservative‐free eyedrops and punctal occlusion. If corneal ulcer develops, a broad spectrum topical antibiotic should be added. Early lateral tarsorrhaphy should be considered for the treatment of extending or nonhealing corneal ulcers.

This case series studying CIP patients has some limitations, including small number of patients due to rarity of the condition, lack of control subjects, and its retrospective nature. Additional studies are needed to determine the natural history and the genotype–phenotype correlation in these disorders.

In conclusion, we present a series of patients with CIP, which in three of them is associated with a novel variant in *PRDM12*. Patients with this genetic variant tend to have more severe ocular involvement than those with *SCN9A* variant. Nonetheless, affected individuals in either group may develop corneal ulcers at an early age. Outcomes tend to be better among patients with the *SCN9A* variant, probably because of some degree of preserved corneal sensitivity compared to patients with *PRDM12* variant. Ocular manifestations in both groups may include varying degrees of neurotrophic keratopathy due to varying degrees of corneal hypoesthesia or anesthesia. Additional studies are needed to quantify the degree of corneal insensitivity and its correlation with severity of ocular involvement.

## CONFLICT OF INTEREST

The authors have no conflicts of interest to declare.

## Data Availability

The data that support the findings of this study are available from the corresponding author upon reasonable request.

## References

[ajmga62968-bib-0001] Chen, Y. C. , Auer‐Grumbach, M. , Matsukawa, S. , Zitzelsberger, M. , Themistocleous, A. C. , Strom, T. M. , Samara, C. , Moore, A. W. , Cho, L. T. Y. , Young, G. T. , Weiss, C. , Schabhüttl, M. , Stucka, R. , Schmid, A. B. , Parman, Y. , Graul‐Neumann, L. , Heinritz, W. , Passarge, E. , Watson, R. M. , … Senderek, J. (2015). Transcriptional regulator *PRDM12* is essential for human pain perception. Nature Genetics, 47(7), 803–808. 10.1038/ng.3308 26005867 PMC7212047

[ajmga62968-bib-0002] Cox, J. J. , Sheynin, J. , Shorer, Z. , Reimann, F. , Nicholas, A. K. , Zubovic, L. , Baralle, M. , Wraige, E. , Manor, E. , Levy, J. , Woods, C. G. , & Parvari, R. (2010). Congenital insensitivity to pain: Novel *SCN9A* missense and in‐frame deletion mutations. Human Mutation, 31(9), E1670–E1686. 10.1002/humu.21325 20635406 PMC2966863

[ajmga62968-bib-0003] Drissi, I. , Woods, W. A. , & Woods, C. G. (2020). Understanding the genetic basis of congenital insensitivity to pain. British Medical Bulletin, 133(1), 65–78. 10.1093/bmb/ldaa003 32219415 PMC7227775

[ajmga62968-bib-0004] Elsana, B. , Gradstein, L. , Imtirat, A. , Yagev, R. , Barrett, C. , Ling, G. , Abu Tailakh, M. , Baidousi, A. , & Tsumi, E. (2021). Ocular manifestations of congenital insensitivity to pain: A long‐term follow‐up. The British Journal of Ophthalmology, 133(1), 317464. 10.1136/bjophthalmol-2020-317464 33753408

[ajmga62968-bib-0005] Kim, D. T. , Rossignol, E. , Najem, K. , & Ospina, L. H. (2015). Bilateral congenital corneal anesthesia in a patient with *SCN9A* mutation, confirmed primary erythromelalgia, and paroxysmal extreme pain disorder. Journal of American Association for Pediatric Ophthalmology and Strabismus, 19(5), 478–479. 10.1016/j.jaapos.2015.05.015 26486037

[ajmga62968-bib-0006] McDermott, L. A. , Weir, G. A. , Themistocleous, A. C. , Segerdahl, A. R. , Blesneac, I. , Baskozos, G. , Clark, A. J. , Millar, V. , Peck, L. J. , Ebner, D. , Tracey, I. , Serra, J. , & Bennett, D. L. (2019). Defining the functional role of Na_V_ 1.7 in human nociception. Neuron, 101(5), 905–19.e8. 10.1016/j.neuron.2019.01.047 30795902 PMC6424805

[ajmga62968-bib-0013] Miller, D. T. , Lee, K. , Chung, W. K. , Gordon, A. S. , Herman, G. E. , Klein, T. E. , Stewart, D. R. , Amendola, L. M. , Adelman, K. , Bale, S. J. , Gollob, M. H. , Harrison, S. M. , Hershberger, R. E. , McKelvey, K. , Richards, C. S. , Vlangos, C. N. , Watson, M. S. , Martin, C. L. , & ACMG Secondary Findings Working Group (2021). ACMG SF v3.0 list for reporting of secondary findings in clinical exome and genome sequencing: a policy statement of the American College of Medical Genetics and Genomics (ACMG). Genetics in Medicine, 23(8), 1381–1390. 10.1038/s41436-021-01172-3 34012068 PMC13097145

[ajmga62968-bib-0007] Mimura, T. , Amano, S. , Fukuoka, S. , Honda, N. , Arita, R. , Ochiai, M. , Yanagisawa, M. , Usui, T. , Ono, K. , Araki, F. , Yamagami, S. , Araie, M. , & Awaya, Y. (2008). In vivo confocal microscopy of hereditary sensory and autonomic neuropathy. Current Eye Research, 33, 940–945. 10.1080/02713680802450992 19085376

[ajmga62968-bib-0008] Miyata, K. , Amano, S. , Sawa, M. , & Nishida, T. (2003). A novel grading method for superficial punctate keratopathy magnitude and its correlation with corneal epithelial permeability. Archives of Ophthalmology, 121(11), 1537–1539. 10.1001/archopht.121.11.1537 14609908

[ajmga62968-bib-0009] Ong Tone, S. , Elbaz, U. , Silverman, E. , Levy, D. , Williams, S. , Mireskandari, K. , & Ali, A. (2019). Evaluation of dry eye disease in children with systemic lupus erythematosus and healthy controls. Cornea, 38(5), 581–586. 30933962 10.1097/ICO.0000000000001902

[ajmga62968-bib-0011] Schon, K. , Parker, A. , & Woods, C. G. (2020. 2018 Feb 8 [Updated 2020 Jun 11]). Congenital insensitivity to pain overview. In M. P. Adam , D. B. Everman , G. M. Mirzaa , et al. (Eds.), GeneReviews® [Internet]. University of Washington, Seattle. Available from: https://www.ncbi.nlm.nih.gov/books/NBK481553/

[ajmga62968-bib-0012] Zhang, S. , Sharif, S. M. , Chen, Y. , Valente, E.‐M. , Ahmed, M. , Sheridan, E. , Bennett, C. , & Woods, G. (2016). Clinical features for diagnosis and management of patients with *PRDM12* congenital insensitivity to pain. Journal of Medical Genetics, 53, 533–535. 10.1136/jmedgenet-2015-103646 26975306 PMC4975812

